# Effect of PUFAs-ω3 and ω6 on oxidative stress of sheep erythrocytes

**DOI:** 10.1186/s12917-025-04762-4

**Published:** 2025-05-10

**Authors:** Valeria Pasciu, Maria Nieddu, Elena Baralla, Ignacio Contreras-Solís, Francesca Daniela Sotgiu, Fiammetta Berlinguer

**Affiliations:** 1 Department of Veterinary Medicine, University of Sassari, Sassari, Italy; 2 Department of Medicine, Surgery and Pharmacy, University of Sassari, Sassari, Italy; 3 Department of Agronomy, Biotechnology and Food, University of Navarre, Pamplona, Spain

**Keywords:** Sheep erythrocytes, PUFAs-ω3, PUFAs-ω6, Oxidative stress, Reactive oxygen species, Antioxidant assays

## Abstract

**Background:**

In recent years, the use of long-chain polyunsaturated fatty acids (PUFA) ω3 and ω6, as food supplements in livestock has increased due to their beneficial properties related to their antioxidant activity. It has been demonstrated however that a high intake of these substances has prooxidant and cell-damaging effects, especially if their circulating concentrations are unbalanced. Starting from these premises, and taking advantage of previous findings, the present study aimed at defining the optimal circulating concentrations and PUFAs ω3/ω6 ratio, to ensure the antioxidant/oxidant balance in sheep RBCs.

**Results:**

All tested concentrations (25–300 µg/mL in PBS) of PUFAs-ω3 after 4 h of treatment on sheep RBCs, showed antioxidant properties with a significant decrease in reactive oxygen species (ROS) versus the control group (CTRL) (*p* < 0.05). Furthermore, ω6 showed an antioxidant effect at low concentrations (25–200 g/mL) but a pro-oxidant effect at the highest concentrations (250 and 300 µg/mL) with a significant increase in ROS production (123.6 ± 2.1 and 131.4 ± 6.5% sloope RFU of CTRL respectively *p* < 0,001), malondialdehyde (MDA) (*p* < 0.01), and haemolysis (*p* < 0.01) versus CTRL group (1.1 ± 0.1%), and, also with a decrease of Trolox equivalent antioxidant capacity (TEAC) (*p* < 0,05). The ratio ω3/ω6 of 1:10 (25/250 µg/mL) and 1:4 (25/100 µg/mL) showed an intracellular ROS level like the CTRL group whereas, the ratio 1:2 (100/200 µg/mL) resulted in a significant decrease in ROS production (62.71 ± 2.31% slope RFU of CTRL, *p* < 0.001) and MDA (*p* < 0.001), with an increase in TEAC (*p* < 0.05), and a decrease haemolysis versus the control group (*p* < 0,01).

**Conclusions:**

Our results showed that a beneficial effect on the oxidative state of sheep RBCs was obtained with in vitro administration of low concentrations of ω6 and with all tested concentrations of ω3. The addition of ω6 at high concentrations leads to an imbalance in the PUFA ω3/ω6 ratio, compromising the oxidative state and viability of the RBCs. The maximum antioxidative effect was found at ω3/ω6 ratio 1:2).

## Introduction

Long-chain polyunsaturated fatty acids omega 3 (PUFAs-ω3) and 6 (PUFAs-ω6) represent one of the most widely used antioxidants. In mammals, the body cannot synthesise them but they must be introduced through diet [1, 2]. PUFA-ω3 and -ω6 differ in the location of the first double bond, starting from the methyl end of the molecule. Linoleic acid (LA) is the predominant PUFA-ω6 in nature and it is found in almost all plants except coconut, cocoa, and palm. The most important PUFA-ω3, alpha-linolenic acid (ALA), is found in green leafy vegetables, seeds of flax, rape, chia, perilla, and walnuts [2]. The main biological processes in which they are involved are vision, growth, brain development and reproduction [1, 2, 6].

PUFAs-ω3 and -ω6 are considered the most susceptible reactive oxygen species (ROS) substrates during oxidative stress (OS) conditions [5]. Nutritional epidemiology studies and clinical trials confirm the beneficial effects of food consumption in -ω3 and -ω6 on preventing coronary artery diseases, stroke and dementia in humans [4]. Moreover, a diet enriched in PUFAs-ω3 can prevent vascular dysfunction via multiple mechanisms, including an antioxidant action through the increased activity of endogenous antioxidant response [6, 7]. PUFA-ω3 has also been shown to positively affect the reproductive system in dairy cattle [8]. In a previous work in Sarda sheep [9] we showed that a diet integrated with by-pass linseed oil (LO) rich in PUFAs-ω3 provided beneficial effects on the function of the ovarian corpus luteum. Moreover, it proved to have a positive impact on antioxidative defences on maternal structures during the embryo-maternal recognition period in ewes particularly on the luteal tissues, with an increase in total antioxidant capacity (TEAC), in the enzymatic activity of antioxidant defences and total thiols [9].

The interaction between -ω3 and -ω6 metabolism in the context of inflammation is not fully understood, but it is thought that PUFAs-ω3 and -ω6 likely work synergistically to produce anti-inflammatory effects [10, 11]. However, it is also necessary to underline that a high intake of PUFAs-ω6 in the diet can shift the physiological state to a proinflammatory and prothrombotic one [12]. The metabolism of ω6 specifically produces eicosanoids such as prostaglandins, thromboxanes, leukotrienes, hydroxy fatty acids, and lipoxins in greater quantity than those derived from PUFAs-ω3 [13]. These products, already biologically active in small amounts, contribute to inflammatory disorders, thrombus formation and allergic processes, particularly in susceptible subjects [14]. These inflammation processes are associated with oxidation phenomena and are implicated in initiating atherosclerosis and endothelial and blood cell dysfunction [11].

In non-human primates and rats, dietary supplementation rich in -ω3 did not modify the ratio of unsaturated/saturated fatty acids in the erythrocyte membranes but only the concentrations of unsaturated fatty acids. In fact, after 12 weeks of -ω3 supplementation, in the RBCs membrane, PUFAs-ω3 increased and PUFAs-ω6 decreased, while concentrations of saturated fatty acids remained unchanged. This alteration of the fatty acid profile of the RBC membranes could have a potential biological impact on the inflammation process, lipid metabolism, and overall health state [15, 16].

Therefore, the balance of PUFAs-ω3 and -ω6 in the diet is crucial because they can have opposing physiological effects. Considering that the mammalian cell cannot convert PUFAs-ω3 into -ω6, due to the lack of the enzyme ω3 desaturase, it is necessary to supply the body with a balanced quantity of these substances [17]. For this reason, many studies investigated the effects of different PUFA ratios in order to identify the optimal dietary intake [18–22].

For example, Ghazali et al. reported that in hepatic cells low ω3/ω6 ratios (1:15, 1:25) damaged mitochondrial functions, increasing ROS production, and reducing mitochondrial respiratory chain and ATP production; on the contrary, at higher ratios (1:1 and 1:4) hepatic cells showed the same mitochondrial functionality as the control [22]. Some researchers have suggested reducing PUFAs-ω6 consumption [18, 23, 24], or increasing ω3 intake [18, 25] to improve the ω3/ω6 ratio and reduce cardiovascular diseases. Furthermore, several authors agree on the ideal ω3/ω6 ratio of 1:1 or 1:2 for treating cancer [18, 26].

Based on these findings, the present study was designed to determine the concentrations of PUFAs ω3, -ω6 and their ratio that would ensure the optimal antioxidant/oxidant balance in sheep red blood cells (RBCs). Oxidative stress in cells results from an imbalance between the level of reactive oxygen species (ROS) and antioxidant defence systems [27, 28]. Since RBCs are naturally exposed to circulating concentrations of PUFAs ω3/ω6 that can vary with the animal’s diet, we investigated the effects of different ratios of PUFAs ω3/ω6 on the redox state of sheep erythrocytes in vitro.

## Results

**Fig. 1:**
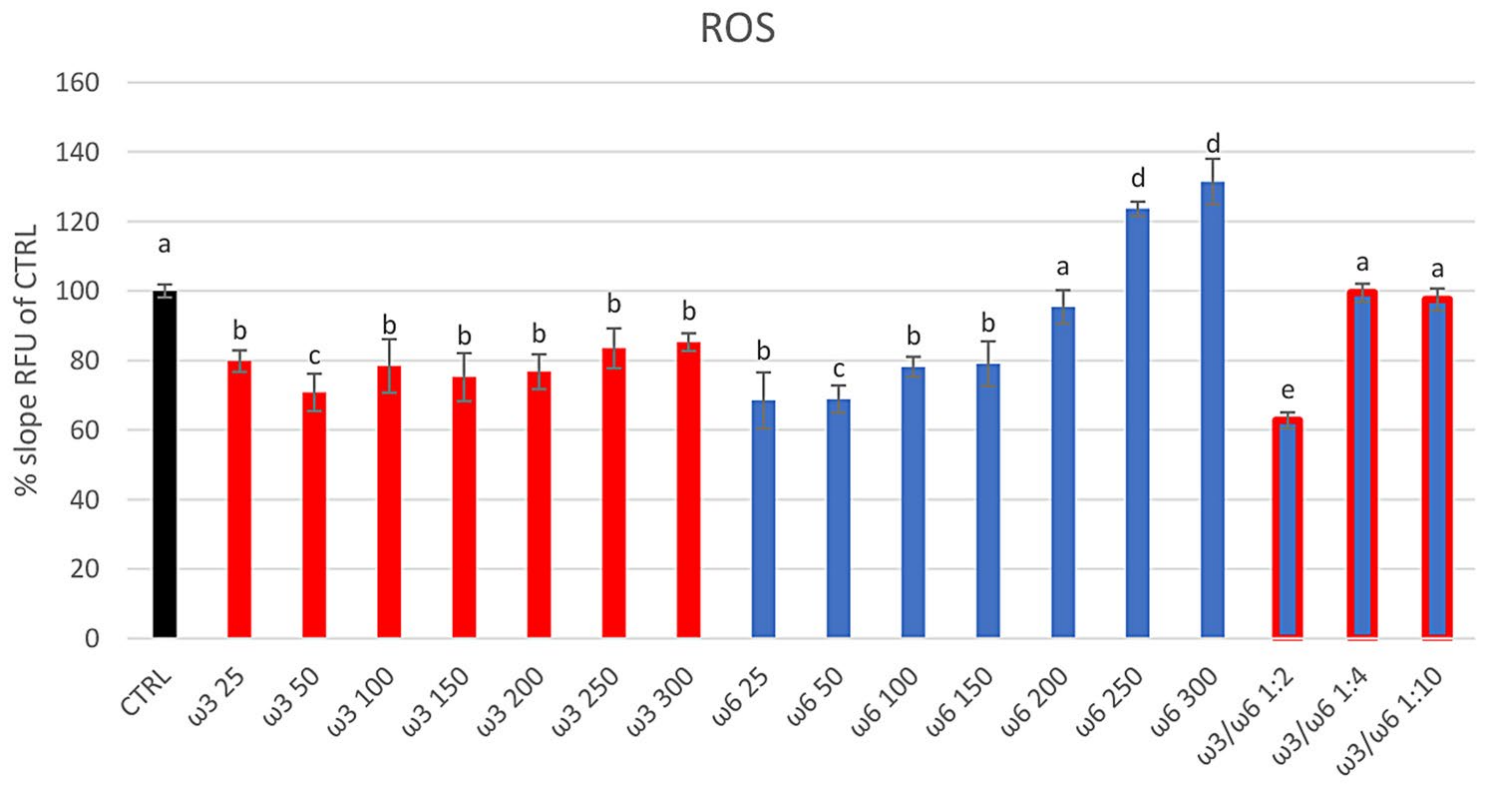
Intracellular ROS production in sheep RBCs using H2DCF-DA and measuring RFU (RFU measurement RFU = Relative Fluorescent Units) production every 10 min for 4 h. RBCs were treated in vitro at 37 °C with PUFAs-ω3 and PUFAs-ω6 at different concentrations individually (25, 50, 100, 150, 200, 250, and 300 µg/mL), and using the ratio ω3/ω6 of 1:2 (100/200), 1:4 (25/100 µg/mL), 1:10 (25/250 µg/mL). Untreated RBCs at 37 °C for 4 h represent the control group (CTRL). Uppercase letters indicate significant statistical differences between groups of RBCs supplemented with different concentrations of PUFAs (red bars indicate groups with added ω3. Blue bars indicate groups with added ω6. Blue/red bars indicate groups with added ω3/ω6 ratio. Black bar indicated CTRL): *p* < 0.05 (one-way ANOVA)

All tested concentrations of PUFAs-ω3 showed antioxidant properties with a significant (*p* < 0.05) decrease in ROS production *versus* CTRL group (Fig. 1). In contrast, -ω6 showed an antioxidant effect at concentrations ≤ 200 µg/mL, and a pro-oxidant effect at the highest concentrations (250 and 300 µg/mL), with a significant (*p* < 0.001) increase of ROS production *versus* CTRL group (123.61 ± 2.08 and 131.45 ± 6.58% slope RFU of CTRL respectively) (Fig. 1).

The combination of PUFAs-ω3/-ω6 at a 1:2 ratio, resulted in a significant decrease in ROS production (62.71 ± 2.31% slope RFU of CTRL) in sheep erythrocytes (*p* < 0.01) vs. CTRL, while lower ratios were not effective in this regard (Fig. 1).

**Fig. 2:**
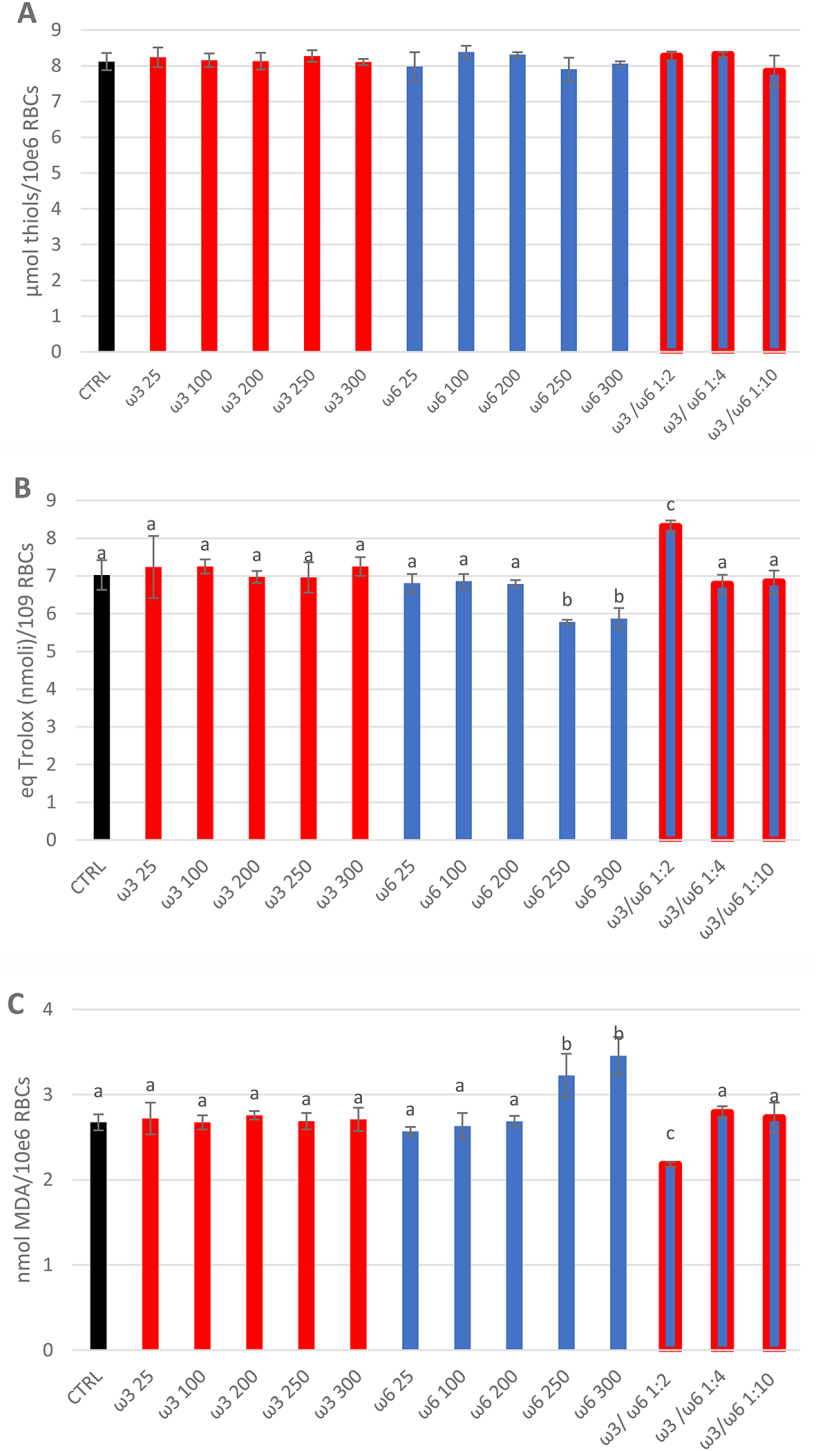
Total thiols (**A**), TEAC (**B**), and MDA (**C**) assay in sheep RBCs treated for 4 h in vitro at 37 °C with PUFAs-ω3 and PUFAs-ω6 at different concentrations individually (25, 100, 200, 250 and 300 µg/mL), and using the ratio ω3/ω6 of 1:2 (100/200 µg/mL), 1:4 (25/100 µg/mL), 1:10 (25/250 µg/mL). Untreated RBCs after 4 h at 37 °C represent the control group (CTRL). Uppercase letters indicate significant statistical differences between groups of RBCs supplemented with different concentrations of PUFAs (red bars indicate groups with added ω3. Blue bars indicate groups with added ω6. Blue/red bars indicate groups with added ω3/ω6 ratio. Black bar indicated CTRL): *p* < 0.05 (one-way ANOVA)

The concentrations of total thiols did not change significantly when compared to the CTRL group for any investigated PUFA concentrations and ω3/ω6 ratios (Fig. 2A). TEAC significantly decreased (*p* < 0.05) with ω6 at 250 and 300 µg/mL (5.78 ± 0.05 and 5.87 ± 0.27 nmol equivalent Trolox/10^6^ RBCs respectively), whereas increased (*p* < 0.05) in the presence of the ω3/ω6 ratio 1:2 (8.31 ± 0.15 nmol equivalent Trolox/10^6^ RBCs) when compared to CTRL (7.02 ± 0.39 nmol equivalent Trolox/10^6^ RBCs) (Fig. 2B). Moreover, MDA levels significantly increased with ω6 at 250 and 300 µg/mL (*p* < 0.01) (3.23 ± 0.25 and 3.46 ± 0.22 nmol MDA/10^6^RBCs respectively) and decreased with the ratio 1:2 (*p* < 0.01) (2.18 ± 0.07 nmol MDA/10^6^RBCs), when compared to CTRL group (2.67 ± 0.09 nmol MDA/10^6^RBCs) (Fig. 2C). For all the other tested concentrations and ratios, no significant changes *versus* CTRL groups in TEAC and MDA levels were found (Fig. 2B and C).

**Fig. 3:**
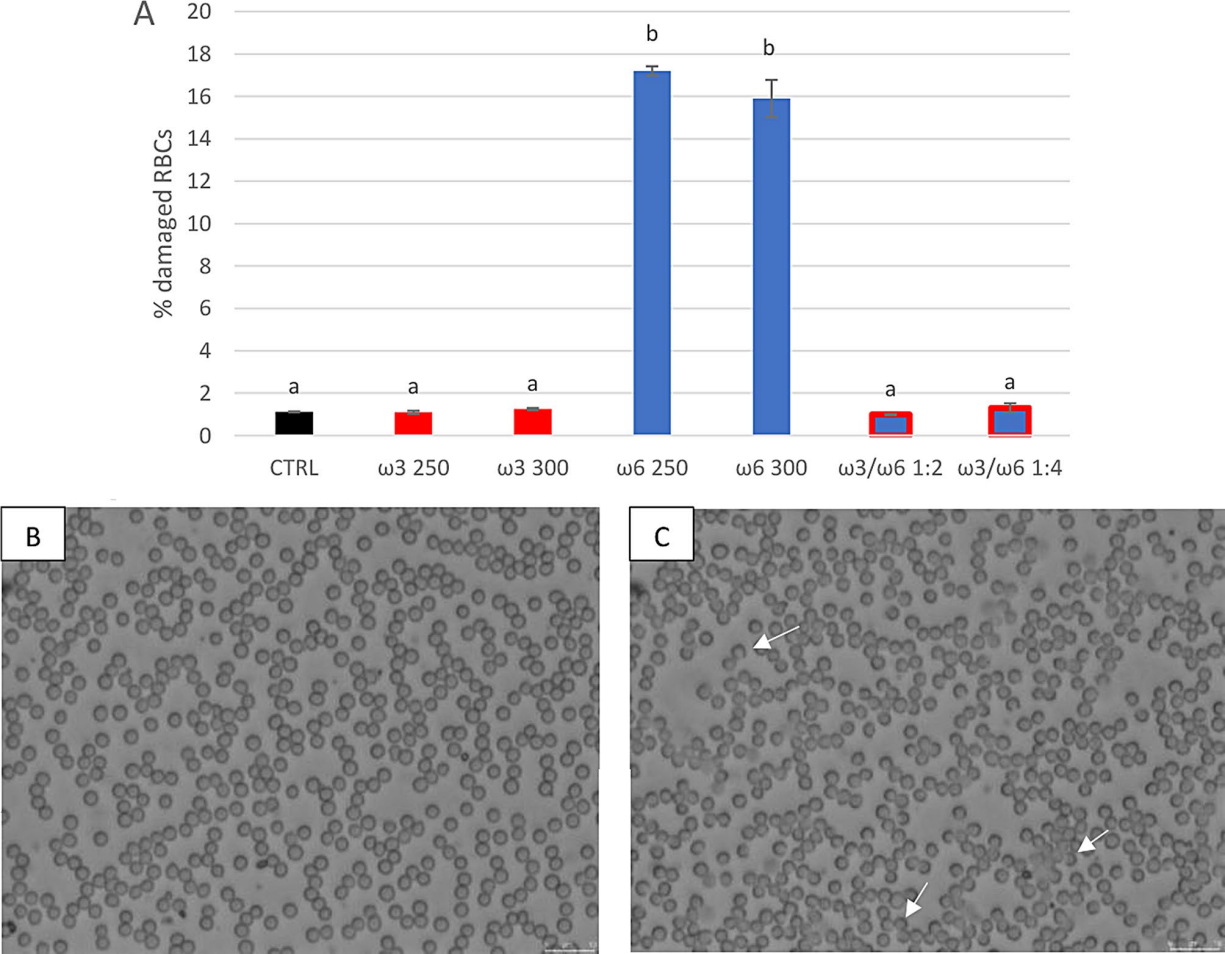
Morphological evaluation of sheep RBCs treated for 4 h in vitro at 37 °C with different concentrations of PUFAs-ω3 and PUFAs-ω6. In Fig. 3A the % damaged RBCs are reported for the CTRL, and the highest concentrations of PUFAs-ω3 and PUFAs-ω6 (250 and 300 µg/mL), and the ratio ω3/ω6 of 1:2 (100/200 µg/mL) and 1:4 (25/100 µg/mL). Uppercase letters indicate significant differences, between groups: *p* < 0.001 (one-way ANOVA). Figure 3B and C show microscopic images of healthy and damaged RBCs, respectively. White arrows indicate examples of damaged erythrocytes (Fig. 3C)

**Fig. 4:**
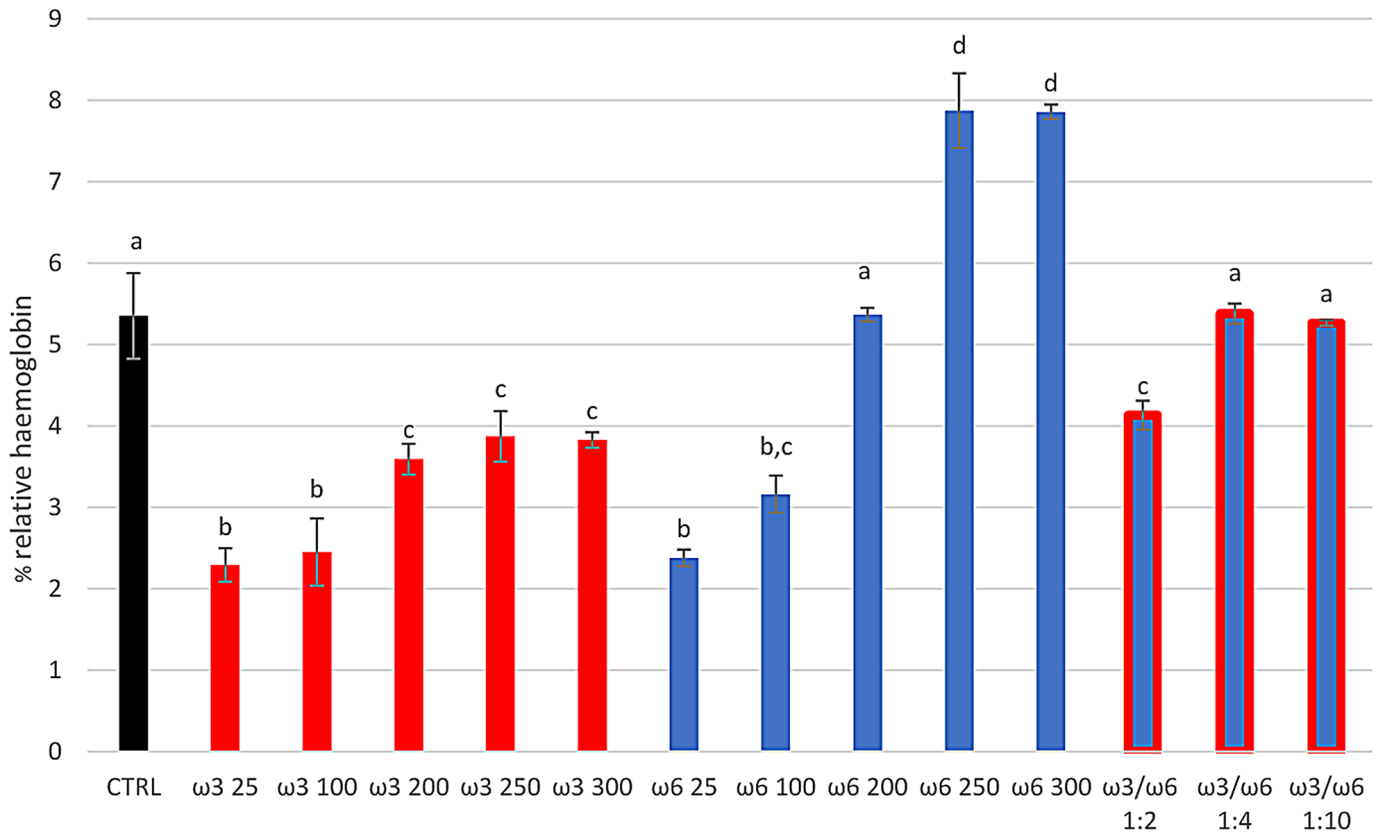
Effect of the different treatments on haemolysis. Relative haemoglobin percentage of sheep RBCs treated for 4 h in vitro at 37 °C with different concentrations of PUFAs-ω3 and PUFAs-ω6 (25, 100, 200, and 250 µg/mL), and using the ratio ω3/ω6 of 1:2 (100/200 µg/mL), 1:4 (25/100 µg/mL), 1:10 (25/250 µg/mL). Untreated RBCs after 4 h at 37 °C represent the control group (CTRL). Uppercase letters indicate significant differences, between groups of RBCs supplemented with different concentrations of PUFAs Uppercase letters indicate significant statistical differences between groups of RBCs supplemented with different concentrations of PUFAs (red bars indicate groups with added ω3. Blue bars indicate groups with added ω6. Blue/red bars indicate groups with added ω3/ω6 ratio. Black bar indicated CTRL): *p* < 0.05 (one-way ANOVA)

Given the intracellular ROS change, both RBCs’ morphological characteristics (Fig. 3) and haemolytic status (Fig. 4) showed significant differences among treated groups.

As shown in Fig. 3, the sheep RBCs treated with the highest concentrations of ω3 (250 and 300 µg/mL) had an aspect similar to the control (% damaged RBCs 1.09 ± 0.08 and 1.25 ± 0.05%, respectively, versus CTRL 1.12 ± 0.03%). In contrast, the corresponding concentrations of ω6 showed a damaged status (% damaged RBCs of 17.2 ± 0.70 and 15.9 ± 0.86%, respectively) when compared to the CTRL (*p* < 0.001). However, when ω6 was combined with ω3 the erythrocytes’ aspect returned like the control (% damaged RBCs of 0.99 and 1.3% for 1:2 and 1:4 ratios, respectively). Moreover, the relative haemoglobin percentage was significantly (*p* < 0.01) lower than CTRL for all ω3 concentrations (50 and 150 µg/mL were not shown), for the lowest ω6 concentrations (from 25 to 150 µg/mL) and ω3/ω6 ratio 1:2. The haemolysis percentage for ω6 200 µg/mL and for ω3/ω6 ratios 1:4 and 1:10 was comparable to the CTRL group (Fig. 4). Instead, the highest ω6 concentrations (250 and 300 µg/mL) showed a significant increase (*p* < 0.01) in the % RBCs haemolysis when compared to the control (Fig. 4).

## Discussion

In recent years, dietary supplementation with ω3 and ω6 has become highly popular among humans and animals [9, 29, 30]. PUFAs-ω3 and -ω6 have demonstrated antioxidant and anti-inflammatory properties [31]. However, several studies have indicated that a diet high in PUFAs-ω6 may lead to a shift from a physiological to prothrombotic and pro-aggregatory state, resulting in increased blood viscosity, vasospasm, and vasoconstriction, and a decreased bleeding time [32, 33]. Furthermore, increased dietary intake of ω6 leads to oxidation of low-density lipoprotein, platelet aggregation, and interferes with the incorporation of essential fatty acids in cell membrane phospholipids [34].

Although in recent years, the consumption of antioxidant substances as supplements to the normal diet has been a diffused trend to reduce the negative effects of OS, a surplus could make them pro-oxidants, generating cell-damaging effects [35–41]. Therefore, it is important to maintain the right ω3/ω6 PUFA ratio in the bloodstream [18, 42, 43].

In a recent paper, a positive antioxidant effect of PUFAs-ω3 on plasma and reproductive tissues (uterus and corpus luteum), after supplementation with linseed oil (rich in PUFAs-ω3), during maternal recognition of the pregnancy period in ewes, has been reported [9]. After this supplementation, the ω3/ω6 ratio in sheep blood plasma was 1:2, *versus* 1:5 for non-supplemented sheep [9]. The authors indicated a protective effect of LO from OS on maternal structures, guaranteeing embryo survival.

Considering that erythrocytes are naturally exposed to circulating concentrations of PUFAs ω3/ω6 and that these concentrations change according to the animal’s diet, we investigated in vitro the effects of different concentrations and different ratios of PUFAs ω3/ω6 on the redox status of sheep erythrocytes.

The results of our work showed that for all studied concentrations of ω3, after 4 h of treatment, there was a decrease in ROS production compared to the control; this is in agreement with what was found in “*in vitro”* cultures for other cell lines [44–46]. In addition, with the same PUFA-ω3 concentrations, TEAC, total Thiols, MDA and RBCs morphology did not change compared to the control. The observed decrease in ROS, with the non-change in the other assayed parameters on our cell model, could suggest a capacity of ω3 to act as an ROS scavenger without changing the concentration of cellular antioxidants, as also reported by Doriane et al. [47] for human aortic endothelial cells.

Moreover, we observed a decrease in haemolysis percentage versus the control with all tested concentrations of PUFA-ω3. The literature reported that by adding fatty acids, the biophysical parameters of cell membranes changed, including their fluidity, and deformability [43, 48]. In particular, Bazzano et al. [49] reported that the supplementation with PUFA-ω3 in horses showed reduced osmotic fragility of erythrocytes and, therefore, greater resistance to haemolysis than horses without supplementation in their diet after show jumping courses. Also, other researchers reported the same effect of PUFA-ω3 on decreased haemolysis in rabbit RBCs [50]. This evidence supports our results for sheep RBCs treated with ω3. However, our results, compared to the literature studies, provide additional information about the different concentrations of ω3 used.

Unlike PUFA-ω3, the -ω6, at the highest concentrations (250 and 300 µg/mL) tested, showed a prooxidant effect on RBCs with a significant increase of ROS production and MDA levels, and a decrease in TEAC *versus* CTRL. This framework suggests the presence of OS with decreased antioxidant defences, increased ROS levels and lipid peroxidation with consequent membrane damage, confirmed by increased haemolysis and alterations in the form and aspect of erythrocytes.

To our knowledge, the effect of ω6 on erythrocytes has not yet been reported. Despite the relationship between PUFAs-ω6 and ROS being poorly investigated in animal cells [51], in humans it has been observed that LA induces ROS production in hepatic cell lines [52, 53] and causes apoptosis, increasing lipid peroxidation in carcinoma cells [52, 54]. Furthermore, Doriane R. et al. [47] reported that supplementation of human aortic endothelial cells with PUFAs-ω3 resulted in a lower production of ROS compared to cells supplemented with PUFAs-ω6.

This trend would confirm what we found in the RBCs and explain the increased haemolysis at high concentrations of -ω6, which acted as a pro-oxidant. Under physiological conditions, the structural alterations linked to oxidative damage lead to the removal of senescent erythrocytes from circulation through an eryptosis process [27]. Under in vitro conditions, as reported by Baralla et al. [55], sheep RBCs, damaged by treatment with pro-oxidant molecules, undergo haemolysis due to membrane damage since they cannot be eliminated by eryptosis.

Several studies also focused on the effect of ω3/ω6 ratio. For example, a decrease of ω3/ω6 ratio was linked to inflammation and chronic diseases [56]. On the contrary, a meta-analysis study indicated that the intake of PUFA-ω3, but not of -ω6 (resulting in a higher ω3/ω6 ratio in the blood), was associated with a lower risk of metabolic syndrome [57]. Also, Park et al. reported the beneficial effect of a higher ω3/ω6 ratio on metabolic dysfunction in mice, inhibiting hepatic triglyceride accumulation and inflammation [58]. Contreras-Solis et al. also report a beneficial effect of a high ratio ω3/ω6 in sheep pregnancy [9].

The results of our study about different ratios ω3/ω6 in sheep erythrocytes confirmed this trend. A protective effect on OS, with a significant decrease in ROS production and MDA and with an increase in TEAC, was found at the 1:2 ratio used. These data were confirmed by a decrease in haemolysis and by RBCs’ morphological aspect.

## Conclusion

The results of our in vitro investigation with sheep RBCs, demonstrated the antioxidant properties of PUFA-ω3 and the prooxidant effect of PUFA-ω6 at high concentrations. However, when ω3 and ω6 were combined in different ratios, the prooxidant effect of ω6 was neutralised, emphasising the importance of monitoring the ω3/ω6 ratio for erythrocyte viability. Our findings confirmed that the in vitro supplementation of ω3/ω6 at a 1:2 ratio, improved the antioxidative/oxidative status of sheep RBCs compared to lower ratios (1:4 and 1:10).

## Materials and methods

### Reagents and animals

All reagents used were purchased from Merck Sigma Aldrich: Fructose (cod: 729051), H_2_DCF-DA (cod: D6883), Linoleic acid (LA) (cod: L8134), alpha-linolenic acid (ALA) (cod: L2376).

All procedures involving animals in this study were approved by the Local Animal Care and Use Committee of the University of Sassari (Authorization code: 2899 of 17/01/2018). Ewes were all owned by the Department of Veterinary Medicine, and they were confined outdoors with access to a sheltered area, at the experimental facilities of the Department of Veterinary Medicine at the University of Sassari, Italy (40°43′40.33″N, 8°33′1.33″E). The experiments were repeated 4 times and each time, blood samples, from four ewes (different ewes each time, aged between 2 and 4 years, average body weight 40–50 kg), were collected at fasting (07:00 a.m.) from the jugular vein using 9 mL vacuum collection tubes containing EDTA K2 (Vacutainer Systems Europe; Becton Dickinson, Meylan Cedex, France). The mean sheep erythrocyte concentration was 12 × 10^9^/mL. The four blood samples were pooled before treatment and analyses.

### Protocol design

Sheep whole blood (pooled blood) was aliquoted and treated with ALA (group ω3, as ALA is the most important PUFA-ω3) and LA (group ω6; as LA is the most important PUFA-ω6) at different concentrations individually (25, 50, 100, 150, 200, 250 and 300 µg/mL), and using the ratio ω3/ω6 of 1:2 (100/200 µg/mL), 1:4 (25/100 µg/mL), 1:10 (25/250 µg/mL) to obtain different treated groups. Every aliquot was incubated at 37 °C for 4 h and used to assay intracellular ROS, total Thiols, Malonyl di Aldehyde (MDA), Trolox Equivalent Antioxidant Capacity (TEAC) and percentage haemolysis as described below; also, the RBCs morphology after treatment was observed. All the assays were performed in quadruplicate. A control group (CTRL) without treatment was incubated for 4 h at 37° C for all experiments.

The concentrations used were chosen based on the PUFAs-ω3 and PUFAs-ω6 circulating concentrations found in sheep after LO administration as a dietary supplement, as described by Contreras-Solís et al. (CTRL PUFAs-ω3 = 0.034 ± 0.014 mg/mL and PUFAs-ω6 = 0.170 ± 0.046 mg/mL PUFAs-ω3/ω6 = 1:5; LO supplemented group PUFAs-ω3 = 0.096 ± 0.034 mg/mL and PUFAs-ω6 = 0.214 ± 0.064 mg/mL PUFAs-ω3/ω6 = 1:2) [9]. In this study, twenty ewes (4.23 ± 0.10 years old Sarda ewes, average body weight 50–52 kg), were fed a diet enriched with microencapsulated LO (18% ALA; SILA™; Verona, Italy), while a separate control group of twenty ewes was fed with an LO-free diet. The experimental period lasted 36 days and concentrations of PUFAs-ω3 and PUFAs-ω6 were determined on day 15 of the experimental phase using Fatty Acids Methyl-Esters (FAMES) analyses [9].

### ROS assay

Blood samples from each treatment were further diluted to 7 × 10^6^ RBCs/mL using PBS plus (PBS with 0,1% fructose) to measure ROS production in erythrocytes. To each treated sample, 2′,7′-dichlorodihydrofluorescein diacetate (H_2_DCF-DA) was added at the final concentration of 3µM. Within the cell, the esterase cleaves the acetate groups on H_2_DCF-DA, thus trapping the reduced form of the probe 2′,7′-dichlorodihydrofluorescein (H_2_DCF). Intracellular ROS oxidise H_2_DCF, yielding the fluorescent product, DCF. After 30 min of incubation at 37 °C, fluorescence was measured using a FLUOstar Omega microplate reader (BMG LABTECH) every 10 min for 4 h (kinetic from t_0_ to t_4_). Excitation and emission wavelengths used for fluorescence quantification were 485 and 535 nm, respectively. All fluorescence measurements (RFU = Relative Fluorescence Unit) were corrected for background fluorescence and replicated four times. Kinetic data were expressed as % slope RFU mean ± SD vs. CTRL [55, 59].

### Total thiols, TEAC, and MDA assays

Blood cells were treated with PBS containing TritonX100 to 0.1% to obtain the cell extract samples for TEAC, MDA, and total thiols assays. These assays were performed as reported in the previous manuscript [27, 55, 60]. All data were normalised for total cells and were assayed using an automatic cell counter instrument (Haematology analyser Alcyon Mindray BC5000, Shenzhen, China).

#### Total thiols

Total Thiols were assayed using the Ellman’s Reagent 5,5-dithiol-bis-(2-nitrobenzoic acid) (DTNB) solved in PBS. Thiols were quantified using a spectrophotometer by measuring the absorbance of visible light at 412 nm, using an extinction coefficient of 14,150 M^− 1^ cm^− 1^ for dilute buffer solutions [35]. The values of Thiols in the samples were expressed in nmol/10^6^ erythrocytes.

#### TEAC assay

TEAC was determined using the method described by Reetal. And modified by Lewinska et al. [61]. Briefly, a fresh solution was prepared by dissolving 19.5 mg 2,20-azinobis (3- ethylbenzthiazoline-6-sulphonic acid [ABTS]) and 3.3 mg potassium persulphate in 7 mL of 0.1 mol/L phosphate buffer, pH 7.4. This solution was stored in the dark for 12 h for the completion of the reaction. ABTS solution was diluted (usually approximately 1:80) in 0.1 mol/L phosphate buffer, pH 7.4, to give an absorbance reading at 834 nm of 1.0 and mixed thoroughly. The antioxidant capacity was expressed as the concentration of Trolox producing the same effect as the sample studied, (TEAC). The values of TEAC in the samples were calculated using a standard curve (5–20 mM Trolox in a total volume of 550 mL) and were expressed as nmoles of Trolox equivalent / Cell extract (nmol TEAC/10^6^ erythrocytes).

#### MDA assay

MDA, one of the several low-molecular-weight end-products of lipid peroxidation, was evaluated by the TBARS assay using Thiobarbituric acid and a spectrophotometric method according to the TBA test described by Spanier and Traylor [62]. The values of MDA in the samples were calculated using a standard curve (2–100 µM) and expressed in nmol/10^6^ erythrocytes.

### RBCs morphological evaluation

After incubation (4 h at 37 °C), blood samples were used to prepare blood smears. A slide for each concentration of PUFAs-ω3 and PUFAs-ω6 and their ratios was viewed at high magnification using an inverted system microscope (Olympus X71 model TH4200). An untreated blood smear was used as the control [55, 60]. RBCs with a well-rounded shape and an intact membrane were considered healthy, while those with damaged membranes and irregular shapes were deemed defective. Morphological evaluation was performed using the Cell* Imaging Software for Life Sciences Microscopy, and the damage was expressed as a percentage of damaged RBCs on a total of 500 RBCs for each treatment group.

### Haemolysis test

After incubation at 37 °C for 4 h with PUFAs-ω3 and -ω6, blood samples were gently centrifuged (100 g for 3 min), and the supernatant was used for the haemolysis test. The test measured the % haemoglobin released from damaged RBCs in the supernatant, reading the absorbance at 405 nm. Data were expressed as means of % relative haemoglobin ± SD. The absorbance value in the supernatant of sheep RBCs lysed in H_2_O was considered 100% haemolysis [55, 60, 63].

### Statistical analysis

Data are expressed as mean ± SEM of at least four replicates. After checking the normality and homogeneity of variances assumptions, differences between experimental groups were analysed by a one-way ANOVA (SigmaPlot 15.0). Statistical significance was accepted at *p* < 0.05.

## Data Availability

The datasets used and/or analysed during the current study are available from the corresponding author on reasonable request.
